# Deep learning models for segmentation and quantification of left atrial appendage volume using noncontrast cardiac computed tomography

**DOI:** 10.1186/s44348-025-00058-1

**Published:** 2025-11-01

**Authors:** Daniel Augusto Message Santos, Lucas de Oliveira Teixeira, Miyoko Massago, Sergio da Alvarez Silva, Sanderland José Tavares Gurgel, Carlos Eduardo Rochitte, Yandre Maldonado e Gomes da Costa, Luciano de Andrade

**Affiliations:** 1 Postgraduate Program in Health Sciences, State University of Maringa, Maringa, Brazil; 2 Postgraduate Program in Computer Science, State University of Maringa, Maringa, Brazil; 3 Department of Medicine, State University of Maringa, Maringa, Brazil; 4 Institute of Heart, State University of São Paulo, São Paulo, Brazil; 5 Department of Informatics, State University of Maringa, Maringa, Brazil

**Keywords:** Atrial appendage, Computed tomography angiography, Deep learning, Artificial intelligence, Diagnostic Image

## Abstract

**Background:**

The left atrial appendage (LAA) is a critical but frequently overlooked site of thrombus formation, reinforcing the need for accurate identification in routine cardiac imaging. This process is related to pathological dilation associated with endothelial injury and a proinflammatory status. This study assesses the performance of deep learning architectures based on U-Net, specifically UNet3D, Residual-UNet3D, 3D Attention-UNet, and Res16-PAC-UNet, in the semiautomated segmentation and volume measurement of LAA.

**Methods:**

We retrospectively analyzed noncontrast cardiac computed tomography (NCCT) scans from 452 patients aged ≥ 60 years, acquired for chest pain evaluation, to compare the performance of four U-Net–based deep learning architectures (UNet3D, Residual-UNet3D, 3D Attention-UNet, and Res16-PAC-UNet) for semiautomated LAA segmentation and volume measurement. Segmentation accuracy was assessed with the Dice coefficient, and volumetric agreement with Pearson correlation and Bland–Altman analysis.

**Results:**

Dice coefficients were 78.44 ± 1.93 for UNet3D, 78.97 ± 0.79 for Residual-UNet3D, 79.07 ± 1.43 for 3D Attention-UNet, and 77.68 ± 1.47 for Res16-PAC-UNet. All models showed strong correlations between predicted and manual volumes (*P* < 0.001), with the highest in 3D Attention-UNet (r = 0.800). Bland–Altman analysis indicated minimal bias and narrow limits of agreement for all architectures, confirming consistent reliability.

**Conclusions:**

Deep learning–based segmentation on NCCT enables accurate, reproducible LAA morphological and volumetric assessment without contrast, offering a rapid and reliable tool to support cardiovascular risk stratification and treatment planning.

**Supplementary Information:**

The online version contains supplementary material available at 10.1186/s44348-025-00058-1.

## Background

The left atrial appendage (LAA) is a critical site for thrombus formation, particularly in conditions involving blood stasis and cardiac dysfunction [1]. Despite its clinical importance, the LAA is often overlooked in routine cardiac imaging, which limits its potential as a risk marker for cardiovascular events [2]. Accurate morphological and volumetric assessment of the LAA is essential but remains challenging due to its anatomical variability and the lack of standardized measurement protocols [3].

Noncontrast cardiac computed tomography (NCCT) provides high-resolution anatomical detail and is commonly used to evaluate coronary artery calcification. However, its use for detailed structural assessment, such as LAA analysis, remains limited [4]. This underutilization is partly due to the lack of specialized automation tools and advanced segmentation software, which reduces the feasibility of incorporating LAA analysis into routine clinical workflows.

In recent years, deep learning has brought significant advances to medical image analysis, particularly in cardiology [5]. Convolutional neural networks (CNNs) have improved the segmentation and quantification of complex cardiac structures such as ventricles, atria, and coronary arteries, which has offered gains in both accuracy and efficiency. Among these, the U-Net architecture has emerged as one of the most effective for semantic segmentation, due to its encoder-decoder structure that balances feature extraction with spatial precision [6].

Variants of U-Net, such as UNet3D, Residual-UNet3D, 3D Attention-UNet, and Res16-PAC-UNet, have demonstrated excellent performance in segmenting complex anatomical structures. Applying these models to NCCT may facilitate more accessible and automated evaluation of LAA morphology and volume, potentially improving risk stratification in clinical cardiology [7, 8].

Despite these promising developments, studies applying deep learning models to NCCT for the purpose of automated LAA segmentation and volume measurement remain scarce. Most of the existing literature has focused on contrast-enhanced coronary CT angiography (CCTA), with limited comparative data for NCCT-based approaches [9–11].

This study aims to validate a U-Net–based deep learning model for semiautomated segmentation and volumetric measurement of the LAA using NCCT images. By facilitating accurate, reproducible assessments without the need for contrast agents, this approach may contribute to earlier detection of LAA dilation and support clinical decision-making in cardiovascular care.

## Methods

### Study design

This retrospective cohort study evaluated the performance of different deep learning architectures for LAA segmentation and volume measurement using NCCT. The study was approved by the Standing Committee on Human Research Ethics of the State University of Maringá (No. 61212222.7.0000.0104), and was conducted in accordance with the CLAIM (Checklist for Artificial Intelligence in Medical Imaging) guidelines [12].

### Study population

We retrospectively selected NCCT scans from patients who underwent chest pain evaluation at a diagnostic imaging center in northern Paraná state, Brazil, between August 2019 and November 2023. Eligible cases were identified through a search of the institutional picture archiving and communication system (PACS) based on a standardized chest pain protocol.

The cohort was restricted to patients aged ≥ 60 years, based on both institutional imaging protocols and a combination of epidemiological and pathophysiological rationale. Large population-based cohort studies, such as the Framingham Heart Study, demonstrate that the incidence of atrial fibrillation approximately doubles with each decade beyond age 60 years [13–17]. This increase in atrial fibrillation burden is accompanied by progressive atrial remodeling, including left atrial enlargement and LAA morphofunctional changes [18, 19], which are most pronounced from the sixth decade onward. Setting the age threshold at ≥ 60 years therefore reduces confounding from developmental and structural heterogeneity, enhances comparability between cases, and targets the population in whom LAA evaluation has the highest clinical relevance whether for thrombus detection, embolic risk stratification, or percutaneous occlusion planning.

Moreover, this approach aligns with international risk stratification tools, such as the CHA_2_DS_2-_VASc (congestive heart failure, hypertension, age ≥ 75 years [doubled], diabetes mellitus, stroke [doubled], vascular disease, age 65–74 years, female sex) score, in which age contributes significantly to thromboembolic risk (1 point for 65–74 years and 2 points for ≥ 75 years) [20]. Although the cutoff used here is slightly below the first scoring threshold, it captures the transitional period where atrial remodeling becomes more prevalent, maintaining epidemiological robustness while expanding the eligible sample size.

Inclusion criteria were as follows: (1) patients aged 60 years or older; (2) clinical diagnosis of unstable angina; (3) creatinine clearance ≥ 30 mL/min/1.73 m2; (4) absence of significant agitation during image acquisition; (5) heart rate ≤ 70 beats per minute at the time of scan; (6) no or mild contrast allergy; and (7) high image quality. Exclusion criteria were applied to the overall imaging protocol, which included both NCCT and CCTA. Patients were excluded if they were aged < 60 years, did not have unstable angina, had a severe contrast allergy (precluding CCTA), presented with low image quality or excessive motion artifacts, or had incomplete clinical data necessary for eligibility verification.

**Fig. 1:**
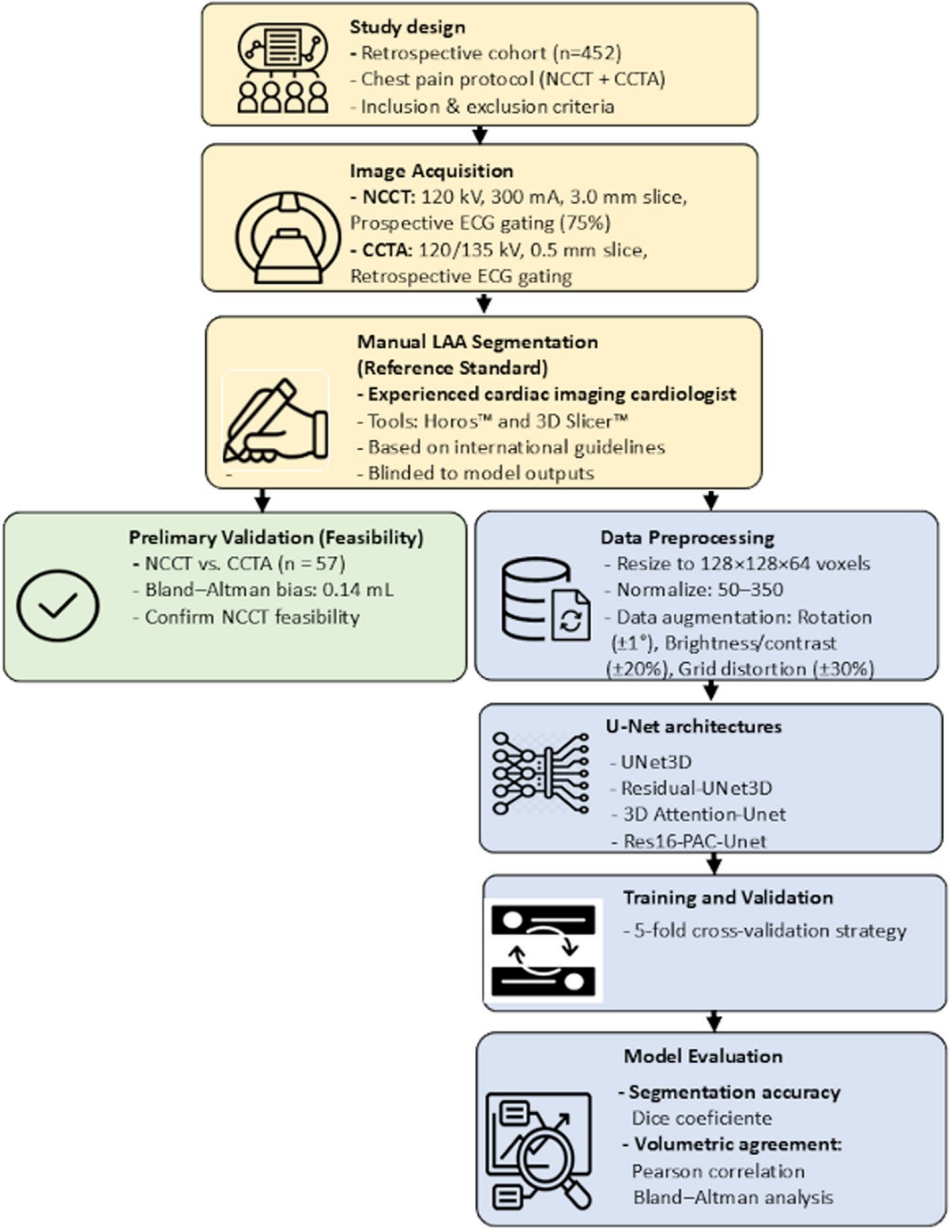
Flowchart illustrates the workflow, which comprises three key components: study design (yellow), preliminary validation (green), and the deep learning pipeline (blue). NCCT, noncontrast cardiac computed tomography; CCTA, coronary computed tomography angiography; ECG, electrocardiogram

Following the application of these criteria, a total of 452 patients were included in the final dataset, each of whom underwent both NCCT and CCTA as part of the standardized chest pain protocol. These paired scans were used for model development and evaluation (Fig. 1).

### Image acquisition

NCCT images were acquired for a preliminary assessment of coronary calcification using an 80-detector tomography scanner (Aquilion Lightning, Canon Medical Systems), with reconstruction in prospective mode and electrocardiographic monitoring at 75% of the cardiac cycle, according to the institution's protocol. This phase corresponds to mid-to-late ventricular diastole. Since the LAA usually reaches its maximal volume during end-systole (atrial diastole) [18, 19], measurements obtained at mid-diastole may slightly underestimate its true maximal volume.

For NCCT, acquisition parameters were tube voltage of 120 kV, tube current of 300 mA, 512 × 512 matrix, FC12 convolution kernel filter, and 3.0-mm slice thickness. CCTA assessment was performed with retrospective electrocardiogram gating using radiation dose modulation (40%–80% according to heart rate parameters). Images were reconstructed at 80% of the cardiac cycle. For CCTA, parameters included tube voltage of 120 kV (135 kV for patients with body mass index > 30 kg/m^2^), 512 × 512 matrix, FC03 convolution kernel filter, and 0.5-mm slice thickness with a 0.3-mm interslice gap.

### Reference annotation (manual segmentation)

Manual segmentation of the LAA was performed by a cardiologist with more than 10 years of experience in both clinical cardiology and advanced cardiovascular imaging, including CCT and cardiac magnetic resonance imaging (MRI). Annotations were conducted in accordance with the “Expert Recommendations on Cardiac Computed Tomography for Planning Transcatheter Left Atrial Appendage Occlusion” [21]. The segmentation protocol specifically excluded the left atrium at the level of the ostium, as well as the pulmonary veins and adjacent anatomical structures, to ensure precise anatomical delineation of the LAA.

Because the CT acquisition protocol was primarily optimized for coronary calcium scoring, the voxel matrix was anisotropic and the slice thickness relatively high. These characteristics limited the accuracy of multiplanar reconstructions and prevented consistent visualization of the LAA ostium in sagittal and oblique planes.

To address this limitation, the boundary between the LAA and the left atrium was defined using standardized internal anatomical criteria, focusing on the axial plane transition zone where the appendage narrows from the atrial body. This approach was applied consistently across all cases by the same cardiologist, in line with recommendations for annotation under suboptimal imaging conditions in cardiac CT [22].

The annotation process was carried out using Horos ver. 3.3.6 (Horos Project) and 3D Slicer ver. 5.0.3 (Slicer Project). Importantly, the expert responsible for the manual annotations was fully blinded to the outputs and quantitative measurements generated by the deep learning models throughout the entire process.

### Preliminary validation between modalities

To verify the feasibility of using NCCT instead of CCTA for LAA volume estimation, we conducted a preliminary comparison in a subset of 57 patients. Bland–Altman analysis showed a minimal mean difference (0.14 mL), indicating that NCCT was sufficiently comparable to CCTA for the purposes of this study.

### U-Net deep learning architectures

U-Net is a widely adopted CNN architecture for medical imaging segmentation tasks [23, 24]. The U-Net consists of an encoder path that captures context and features from the input image, followed by a decoder path that upsamples the encoded features to generate a segmentation map [6]. It leverages skip connections between the encoder and decoder, enabling the model to utilize both local and global information for precise segmentation. We trained and compared four variants: UNet3D, Residual-UNet3D, 3D Attention-UNet, and Res16-PAC-UNet [25–29].

UNet3D extends the standard U-Net architecture to process volumetric medical images, such as CT and MRI scans, by replacing 2D operations with 3D convolutions. This allows the model to better capture spatial dependencies across slices, improving segmentation consistency in 3D structures.

Residual-UNet3D extends the architecture by incorporating residual connections, also known as skip connections, allowing the data to bypass certain layers in the CNN, improving the flow of information and addressing the issue of vanishing gradients in very deep models.

3D Attention-UNet introduces attention mechanisms that selectively weight the importance of different features within the network, allowing the model to focus on more relevant regions during segmentation, which in turn enables the inclusion of finer details, especially in cases where precise localization is important.

Res16-PAC-UNet incorporates multiple pathways with different resolutions, capturing multiscale information, which is particularly useful when dealing with objects of varying sizes, as it allows the model to analyze and incorporate information from different scales simultaneously.

### Model training and validation

Prior to model training, CT images were preprocessed to standardize resolution and intensity across the dataset. Specifically, CT volumes were resampled to 128 × 128 × 64 voxels using spline interpolation along the Z-axis. This resolution was chosen to balance anatomical detail with the computational feasibility of training 3D deep learning models on a single graphics processing unit (GPU; GeForce RTX 3090 Ti, Nvidia Corp). Manual reference segmentations were performed on original-resolution images and subsequently resampled, preserving anatomical precision during training. Voxel intensities were normalized to a window of 50 to 350 Hounsfield units, a standard soft tissue range in NCCT. While this range may exclude hyperdense thrombi or calcifications, the objective of the study was the anatomical segmentation and volume quantification of the LAA, not the detection or classification of intraluminal contents.

Data augmentation was applied using the volumentations library to enhance model generalizability. The transformations included random rotations (between –1° and + 1° along the X-, Y-, and Z-axis), brightness and contrast adjustments (within ± 20%), and grid distortion (up to ± 0.3), each applied with a 50% probability, following parameters adapted from Solovyev et al. [30].

A fivefold cross-validation strategy was employed at the patient level, ensuring that scans from the same individual were not simultaneously included in both training and validation sets. The dataset of 452 CT scans was randomly divided into five equal folds; in each iteration, four folds were used for training and one for validation.

In each fold, the model was trained for 100 epochs with a mini-batch size of 8. The Adam optimizer was used with a fixed learning rate of 0.001. The loss function was the soft Dice loss, which is well suited for volumetric medical image segmentation because it directly optimizes the overlap between predicted and reference masks.

The final performance metrics reported in this study, including Dice coefficients, correlation values, and agreement analyses represent the average results obtained across the five validation folds. No separate holdout test set was reserved in order to optimize the use of the entire dataset during model development. This decision was based on the limited sample size and the exploratory nature of the study.

Once trained, inference was performed on a workstation equipped with an AMD Ryzen 3900X processor (Advanced Micro Devices), 64 GB DDR4 RAM, and a GPU (GeForce RTX 3090 Ti). The average inference time was approximately 2 s per case, enabling rapid mask generation. This represents a substantial efficiency gain compared to manual segmentation workflows, which typically require 10 to 20 min per case depending on image quality and anatomical complexity [29]. For the dataset of 452 cases, automated segmentation reduced the total processing time from an estimated 75 to 150 h (manual) to less than 20 min. These findings highlight the potential for real-time or near-real-time deployment of segmentation models in clinical practice and large-scale imaging studies.

All models were implemented in PyTorch and follow a 3D U-Net–type encoder-decoder design for volumetric segmentation. Each network consists of a contracting path with four downsampling stages and an expansive path with three upsampling stages, ending with a final 1 × 1 × 1 convolution to produce the segmentation map. The standard 3D U-Net applies two consecutive 3 × 3 × 3 convolutions in every block, each followed by batch normalization, a rectified linear unit activation, and dropout with a rate of 0.2. The Residual-UNet3D extends this structure by introducing residual connections that either feed or skip the convolutional layers, improving gradient flow and training stability. The 3D Attention-UNet further augments the architecture by adding spatial and channel attention gates (SCA3D modules) to the skip connections, allowing the network to focus on salient regions of interest. Finally, the Res-PAC-UNet incorporates multiscale pyramid atrous convolutions with dilation rates of 6, 12, and 18 to enlarge the receptive field and capture multiscale contextual information. All models use 32 filters in the first layer, doubling the number of filters after each downsampling step. Downsampling is performed with 2 × 2 × 2 max pooling, and upsampling with transposed convolutions of stride 2. A comprehensive summary of model architectures and training hyperparameters is provided in Supplementary Table 1 to facilitate reproducibility.

### Evaluation metrics and agreement analysis

Model performance was assessed using two complementary approaches: segmentation accuracy and volumetric agreement. The Dice coefficient was calculated to quantify the spatial overlap between the predicted segmentation masks and the manual reference annotations, serving as the primary metric for segmentation accuracy. Comparisons of Dice coefficients across the four deep learning architectures were performed using the Friedman test, followed by pairwise Wilcoxon signed rank tests with Bonferroni correction for multiple comparisons. Statistical significance was set at *P* < 0.05.

For volumetric analysis, LAA volume was calculated for both the predicted and reference masks by multiplying the total number of voxels in each mask by the volume of a single voxel, derived from the original Digital Imaging and Communications in Medicine (DICOM) metadata (voxel spacing in the x, y, and z directions). Although segmentation masks were generated from resampled images at 128 × 128 × 64 resolution, all volume calculations were performed using the original voxel dimensions from the source CT scans to ensure anatomical accuracy.

The relationship between predicted and reference volumes was assessed using the Pearson correlation coefficient, quantifying the strength of the linear association between the two methods. Agreement was further evaluated through Bland–Altman analysis, which estimated the mean bias and calculated the 95% limits of agreement, providing insight into systematic differences and measurement variability.

All statistical analyses were performed using R ver. 4.3.2 (R Foundation for Statistical Computing).

**Fig. 2:**
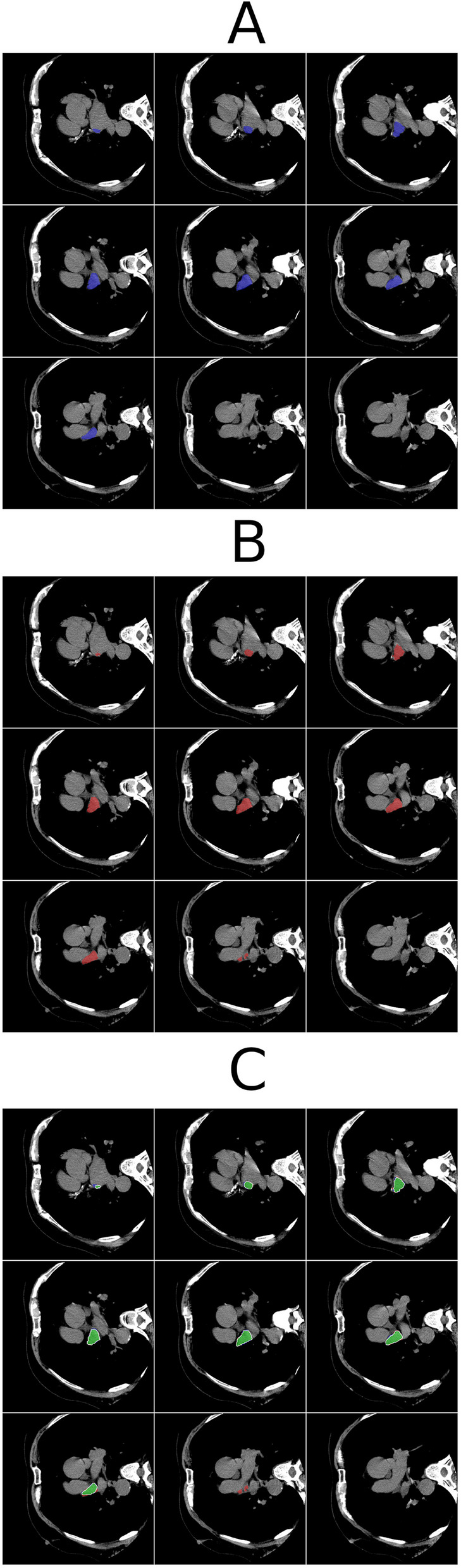
Representative axial slices from a single patient illustrating segmentation of the left atrial appendage across three consecutive levels. **A** Expert manual reference annotation in blue. **B** Prediction by the 3D Attention-UNet model in red. **C** Residual-UNet3D segmentation, with green indicating overlap between model output and ground truth, blue indicating ground truth only, and red indicating model-only regions. Overlays are shown on high-resolution noncontrast cardiac computed tomography images to enhance visual clarity and enable detailed comparison of anatomical boundaries. The same axial positions are presented in all panels to facilitate direct evaluation of spatial accuracy and contour agreement between expert and model-derived segmentations. Colors were selected to maximize contrast against background anatomy, and annotations were verified in both axial slices and 3D volumetric reconstructions during model evaluation

This combined evaluation framework, incorporating both spatial and volumetric metrics, enabled a comprehensive assessment of the accuracy, consistency, and potential clinical reliability of the segmentation models. An illustrative example of the segmentation outputs is presented in Fig. 2, demonstrating the spatial agreement and discrepancies between manual reference annotations and predictions from the evaluated U-Net architectures. The figure displays nine representative axial CT slices from the same patient, with each panel corresponding to different segmentation sources, enabling side-by-side visual comparison of anatomical delineation accuracy.

## Results

This study analyzed data from 452 patients, with a median age of 68 years (interquartile range, 63–73 years), and 282 (62.4%) were male. The median body mass index was 28 kg/m^2^ (interquartile range, 26–32 kg/m^2^), and the most prevalent comorbidities were hypertension (80.3%), dyslipidemia (40.7%), and type 2 diabetes (36.1%). A summary of the clinical and demographic characteristics is presented in Table 1.

**Table 1:** Clinical and demographic characteristics of the study population (*n* = 452)

Characteristic	Value
Age (yr)	68 (63–73)
Sex	
Male	282 (62.4%)
Female	170 (27.6%)
Body mass index (kg/m^2^)	28 (26–32)
Comorbidity	
Hypertension	363 (80.3%)
Type 2 diabetes	163 (36.1%)
Dyslipidemia	184 (40.7%)
Heart failure	31 (6.9%)
Cardiovascular disease	107 (23.7%)
Smoking	56 (12.4%)

Values presented as median (interquartile range) or number (%). The clinical variables were not used in model training or evaluation, and no intergroup comparisons were conducted, as this was beyond the study’s primary methodological focus

**Fig. 3:**
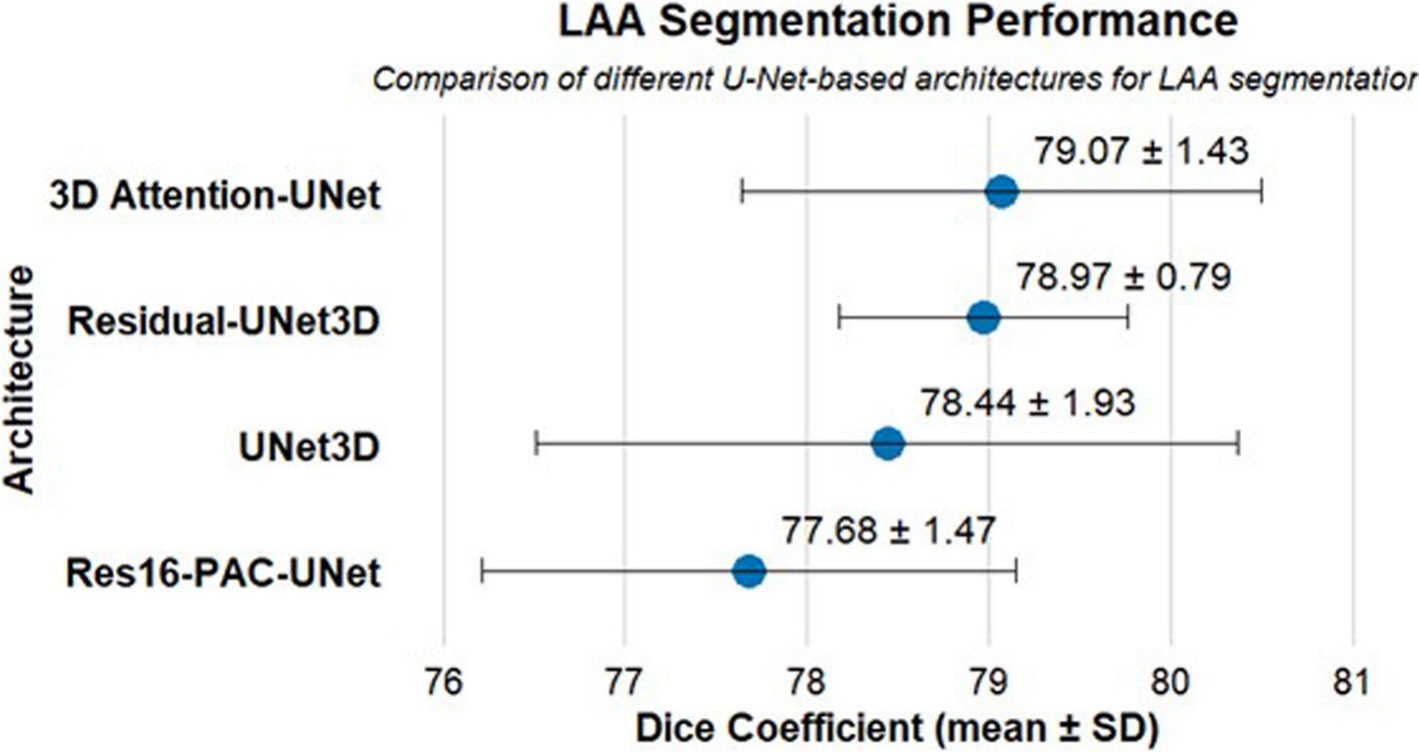
Dice coefficients for left atrial appendage (LAA) segmentation across four deep learning architectures. Error bars indicate standard deviation (SD). Values are presented as mean ± SD

The segmentation performance of the four deep learning models was evaluated using the Dice similarity coefficient (Fig. 3). Among the models, the 3D Attention-UNet achieved the highest Dice score (79.07 ± 1.43), indicating the greatest spatial agreement with the manual reference annotations. The Residual-UNet3D also exhibited strong performance (78.97 ± 0.79), characterized by a slightly lower mean Dice but the smallest standard deviation, reflecting highly consistent segmentation across the dataset. The UNet3D yielded a Dice coefficient of 78.44 ± 1.93, while the Res16-PAC-UNet presented the lowest performance (77.68 ± 1.47), though still within an acceptable range for volumetric segmentation tasks.

A Friedman test comparing Dice coefficients across the five cross-validation folds for all architectures showed no statistically significant difference (χ2 = 7.80, *P* = 0.0503). Post hoc Wilcoxon signed rank tests with Bonferroni correction confirmed that no pairwise comparison reached statistical significance (all adjusted *P* ≥ 0.375). These findings indicate that the segmentation performance of the four architectures was statistically comparable, despite small numerical differences in mean Dice scores. Overall, the relatively low standard deviations, particularly for the Residual-UNet3D and 3D Attention-UNet, indicate consistent segmentation performance across the dataset, with minimal variability between folds.

Following the spatial segmentation analysis based on the Dice similarity coefficient, we evaluated the volumetric consistency of the models, as accurate volumetric estimation is essential for risk assessment and procedural planning. Pearson correlation analysis confirmed a strong linear relationship between the predicted LAA volumes and the reference manual measurements. All models exhibited statistically significant correlations (*P* < 0.001), with correlation coefficients ranging from 0.762 to 0.800. The 3D Attention-UNet achieved the highest correlation (r = 0.800), reflecting the strongest agreement in volumetric estimation, whereas the Residual-UNet3D presented the lowest correlation (r = 0.762).

**Fig. 4:**
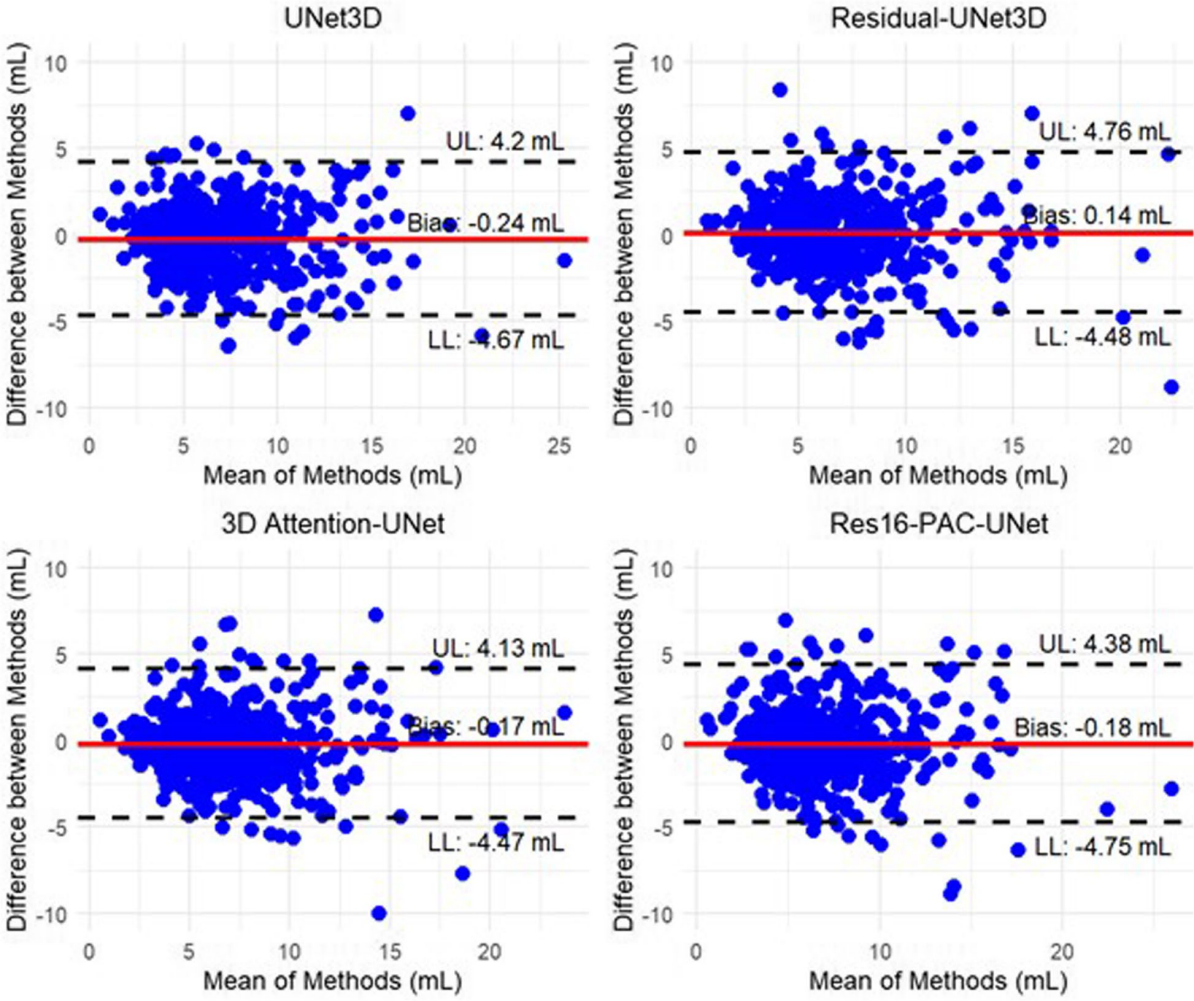
Bland–Altman plots comparing left atrial appendage volumes predicted by deep learning models with reference manual segmentations, illustrating mean bias and limits of agreement. **A** UNet3D. **B** Residual-UNet3D. **C** 3D Attention-UNet. **D** Res16-PAC-UNet. UL, upper limit; LL, lower limit

To further assess agreement and potential bias between the automated and reference manual LAA volume measurements, we performed Bland–Altman analysis (Fig. 4). The mean differences (bias) in LAA volume were found to be –0.24 mL for U-Net3D, 0.14 mL for Residual-UNet3D, –0.17 mL for 3D Attention-UNet, and –0.18 mL for Res16-PAC-UNet. These results indicate a good agreement between the deep learning models and the ground truth, with minimal bias across all methods. The limits of agreement suggest that the variability between methods is relatively low, supporting the reliability of the deep learning segmentations for LAA volume estimation.

## Discussion

To the best of our knowledge, this study represents the first to demonstrate the feasibility and performance of deep learning models for the automatic segmentation and accurate volume quantification of the LAA directly from NCCT scans. The proposed U-Net-based architectures achieved high spatial and volumetric agreement with expert manual annotations, underscoring their significant potential for accelerating and standardizing LAA analysis within clinical workflows.

The evaluated U-Net variants, particularly the 3D Attention-UNet (Dice score, 79.07 ± 1.43) and Residual-UNet3D (Dice score, 78.97 ± 0.79), demonstrated robust segmentation performance with low standard deviations. These Dice coefficients stand out, especially considering the challenges inherent in LAA segmentation, which include its significant anatomical variability, complex contours, and thin walls, and the absence of intravenous contrast in NCCT imaging [30, 31]. While larger, more homogeneous cardiovascular structures typically yield Dice values exceeding 0.90, reported Dice coefficients for smaller, irregular atrial chambers often range between 0.60 and 0.70 [32, 33]. Our results, ranging from 0.77 to 0.79, are consistent with the literature for challenging structures and reflect a high level of anatomical fidelity [34].

Furthermore, the average inference time of approximately 2 s per case represents a more than 60-fold reduction compared to expert manual segmentation, suggesting the potential for real-time or near-real-time analysis without compromising accuracy.

Beyond spatial accuracy, the models also demonstrated excellent volumetric agreement. Pearson correlation analysis confirmed a strong linear relationship between the automated and manual LAA volume measurements across all models, with coefficients ranging from 0.762 to 0.800 (*P* < 0.001). The 3D Attention-UNet consistently showed the highest concordance (r = 0.800).

Complementary Bland–Altman analysis further supported this agreement, revealing minimal mean biases (range, − 0.24 to + 0.14 mL) and narrow limits of agreement. This level of precision is clinically significant, particularly when compared to a prior study that reported considerably higher underestimations, such as a mean underestimation of 5.86 mL/m^2^ for left atrial segmentation in NCCT using other variational autoencoder models [35]. Our models' significantly lower deviations underscore the reliability of U-Net-based architectures for precise LAA quantification.

From a clinical standpoint, the ability to accurately quantify LAA volume using NCCT is highly relevant for clinical decision-making and treatment planning. LAA enlargement is a known risk factor for atrial fibrillation and thromboembolism. The semiautomated approach validated in this study offers a noninvasive and efficient method for assessing LAA morphology and volume, which could facilitate earlier detection of LAA dilation and improve risk stratification in cardiovascular care. Future research may benefit from integrating patient-level clinical data (e.g., rhythm status, atrial size indices) into model pipelines, enabling a more comprehensive and personalized cardiovascular risk assessment.

## Limitations

This study presents certain limitations. First, its retrospective single-center design, with all scans acquired using CT scanners from the same vendor and protocol, may limit generalizability to other settings. Second, LAA segmentation and volumetric measurements were performed at mid-diastole (approximately 75% of the R-R interval), whereas end-systole is typically preferred for maximal volume assessment; this timing difference may have led to underestimation. Third, the absence of an independent holdout set and external validation may slightly overestimate model performance. Future studies with external validation in independent cohorts are essential to confirm the reproducibility and clinical utility of our approach.

In addition, this study focused solely on morphological and volumetric assessment of the LAA, without addressing thrombus detection. Further investigations are needed to evaluate whether NCCT-based deep learning methods could also assist in identifying thrombus and potentially reduce the reliance on contrast-enhanced imaging. Moreover, studies specifically including patients with left atrial enlargement will be necessary to further explore the applicability of LAA volume quantification in this higher-risk subgroup. Finally, only patients aged ≥ 60 years were included, restricting applicability to younger populations.

Nevertheless, our findings demonstrate that deep learning U-Net architectures can achieve accurate, rapid, and reproducible LAA segmentation and volumetric quantification directly from NCCT scans. By facilitating precise and reproducible assessments without the need for contrast agents, this approach may contribute to the earlier detection of LAA dilation and support clinical decision-making in cardiovascular care. The combination of strong spatial and volumetric agreement with expert annotations, coupled with substantial time savings compared with manual segmentation, reinforces the potential clinical applicability of these methods despite the acknowledged limitations.

## Conclusions

Our study employed deep learning U-Net architectures for accurate LAA volume measurement from NCCT. Given the LAA's critical role in thrombus formation and the imperative for its precise identification in routine imaging, these robust models offer an efficient tool for clinical workflows. Further multicenter trials are essential to establish broad generalizability and confirm correlations with future clinical outcomes.

## Supplementary Information

Supplementary Material 1. Table S1. Model architectures and training hyperparameters for reproducibility.

## Data Availability

The dataset(s) supporting the conclusions of this article is(are) available in the Figshare repository, [https://figshare.com/account/articles/26304478?file = 47693425](https:/figshare.com/account/articles/26304478?file = 47693425).

## Abbreviations

CCTA: Coronary computed tomography angiography
CHA_2_DS_2_ -VASc: Congestive heart failure (1), Hypertension (1), Age ≥75 years (2), Diabetes mellitus (1), Stroke (2), Vascular disease (1), Age 65–74 years (1), Sex category female (1)
CLAIM: Checklist for Artificial Intelligence in Medical Imaging
CNN: Convolutional neural network
CT: Computed tomography
DICOM: Digital Imaging and Communications in Medicine
GPU: Graphics processing unit
LA: Left atrium
LAA: Left atrial appendage
MRI: Magnetic resonance imaging
NCCT: Noncontrast cardiac computed tomography
PACS: Picture archiving and communication system
