# Supplementary material for: Deep learning models for segmentation and quantification of left atrial appendage volume using noncontrast cardiac computed tomography
Source: J Cardiovasc Imaging. 2025 Nov 1;33:16. doi: 10.1186/s44348-025-00058-1 (PMC12579425; doi:10.1186/s44348-025-00058-1)
Supplement: Supplementary file 1 — Supplementary Material 1. Table S1. Model architectures and training hyperparameters for reproducibility. [file 44348_2025_58_MOESM1_ESM.docx]

# Supplementary Material

Table S1. Model architectures and training hyperparameters for reproducibility

| Model | Architecture details | Optimizer | Learning rate | Batch size | Epochs | Loss function |
| --- | --- | --- | --- | --- | --- | --- |
| UNet3D | 3D encoder–decoder with 4 downsampling and 3 upsampling stages; two 3×3×3 convolutions per block; batch normalization; ReLU; dropout 0.2; 32 filters in first layer, doubling at each stage; 2×2×2 max pooling; transposed convolutions for upsampling | Adam | 0.001 | 8 | 100 | Soft Dice loss |
| Residual-UNet3D | 3D encoder–decoder with 4 downsampling and 3 upsampling stages; two 3×3×3 convolutions per block; batch normalization; ReLU; dropout 0.2; 32 filters in first layer doubling at each stage; 2×2×2 max pooling; transposed convolutions for upsampling; plus residual skip connections to improve gradient flow and stability. | Adam | 0.001 | 8 | 100 | Soft Dice loss |
| 3D Attention-UNet | 3D encoder–decoder with 4 downsampling and 3 upsampling stages; two 3×3×3 convolutions per block; batch normalization; ReLU; dropout 0.2; 32 filters in first layer doubling at each stage; 2×2×2 max pooling; transposed convolutions for upsampling; plus spatial and channel attention gates (SCA3D modules) on skip connections to highlight salient regions. | Adam | 0.001 | 8 | 100 | Soft Dice loss |
| Res-PAC-UNet | 3D encoder–decoder with 4 downsampling and 3 upsampling stages; two 3×3×3 convolutions per block; batch normalization; ReLU; dropout 0.2; 32 filters in first layer, doubling at each stage; 2×2×2 max pooling; transposed convolutions for upsampling; with pyramid atrous convolutions using dilation rates of 6, 12, and 18 to enlarge receptive field and capture multi-scale contextual. | Adam | 0.001 | 8 | 100 | Soft Dice loss |

## Additional notes

• Implementation: All models were implemented in PyTorch (version X.X).

• Data preprocessing: Volumes resampled to 128 × 128 × 64 voxels; intensity normalized to 50–350 HU.

• Data augmentation: Random rotations (±1°), brightness/contrast adjustments (±20%), and grid distortion (±0.3), each applied with 50% probability.

• Hardware: Training performed on an NVIDIA RTX 3090 Ti GPU, AMD Ryzen 3900X CPU, and 64 GB DDR4 RAM.

• Inference speed: ~2 seconds per case, compared to 10–20 minutes for manual segmentation.
